# Cultivation mechanism of college students’ ethical judgment in the post-truth era: a dual-dimensional model based on critical thinking and emotional education

**DOI:** 10.3389/fpsyg.2026.1798907

**Published:** 2026-04-16

**Authors:** Yaqiong Ding, Yongwen Jiang

**Affiliations:** Faculty of Education, Yunnan Normal University, Kunming, China

**Keywords:** critical thinking, dual-dimensional model, emotional education, ethical judgment, ethical judgment of college students, post-truth

## Abstract

In the post-truth era, the widespread use of social media and algorithmic recommendation technology has gradually made the influence of emotions and beliefs surpass objective facts. College students are particularly vulnerable to emotional and one-sided information, posing severe challenges to their ethical judgment. To address this issue, this study proposes and validates a dual-dimensional model based on critical thinking and emotional education. The aim is to systematically enhance college students’ ethical judgment through collaborative intervention in cognitive and emotional dimensions. The study adopts a randomized controlled experiment design, selecting 600 college students to be divided into a dual-dimensional intervention group, a single-dimensional intervention group, and a control group. The effects are evaluated through pre-tests, post-intervention tests, and follow-up tests. The results show that the ethical judgment of the dual-dimensional intervention group significantly improves compared to the single-dimensional intervention group, and emotional education uniquely contributes to the emotional dimension of ethical judgment. Furthermore, post-truth sensitivity negatively moderates the intervention effect, indicating that individuals with a higher reliance on emotional information benefit relatively limitedly. This study not only fills the gap in ethical education research in the post-truth environment but also provides a modular and quantifiable intervention program for university curriculum design, possessing significant theoretical value and practical implications.

## 1 Introduction

Contemporary society has fully entered the post-truth era, where the influence of emotions and beliefs has gradually surpassed objective facts, becoming the core driving force behind public cognition and decision-making (Viyanti et al., 2025). This phenomenon is particularly pronounced among college students. The widespread use of social media and algorithm recommendation technology has made it easier for emotional and one-sided information to spread, while rational analysis based on facts has become increasingly marginalized. As the backbone of future society, the shaping of college students’ ethical judgment not only concerns their personal development but also directly affects the direction of social moral trends (Wedana et al., 2025; Wang, 2025). However, the current education system still shows deficiencies in addressing the challenges of post-truth. Traditional ethical education places too much emphasis on theoretical indoctrination and lacks comprehensive cultivation of emotional factors and critical thinking. Against this backdrop, how to enhance college students’ ethical judgment through scientific educational intervention has become a major issue of common concern in education, psychology, and sociology (Zondo et al., 2025).

This study aims to explore effective cultivation mechanisms for college students’ ethical judgment in the post-truth era, focusing on the synergistic effect of critical thinking and emotional education (Fabio et al., 2025). Compared with existing research, the theoretical innovation of this study lies in the first proposal and verification of the practical value of the “dual-dimensional model,” which systematically enhances ethical judgment through joint intervention in cognitive and emotional dimensions (Holben et al., 2025; Stavropoulou et al., 2025). At the methodological level, this study adopts a rigorous randomized controlled trial design to ensure the scientificity and generalizability of research conclusions. At the practical level, the intervention curriculum package developed in this study is modular and quantifiable, providing direct reference for the reform of ethical education in universities. By combining theoretical construction and empirical testing, this study attempts to fill the gap in ethical education research in the post-truth environment and provide a basis for the formulation of relevant policies (Haider and Habiba, 2025).

The academic value of this study lies in deepening the understanding of the formation mechanism of ethical judgment, especially revealing the dynamic interaction between cognitive and emotional factors in the post-truth environment (Zuo and Wen, 2025). From a social perspective, the research findings will help optimize the design of ethical courses in the higher education system and cultivate talents for the new era with a more rational spirit and a stronger sense of social responsibility. Furthermore, the dual-dimensional intervention model proposed in this study can be extended to areas such as media literacy education and civic moral construction, providing new ideas for addressing the social governance challenges in the post-truth era (Bartolome and Miranda, 2025).

This study makes three contributions to the literature on ethical judgment in higher education. First, it extends existing research from a single-factor perspective to a two-dimensional framework that conceptualizes ethical judgment as the joint outcome of cognitive discipline and affective engagement, rather than as a purely rational or purely emotional process. Second, it introduces post-truth sensitivity as a boundary condition and shows that the effectiveness of educational intervention is shaped by students’ tendencies to rely on emotionally charged information. In this sense, the proposed model is not only explanatory but also condition-sensitive. Third, the study translates this integrated framework into an empirically testable educational design, thereby linking conceptual discussion on ethical judgment with intervention-based evidence from the university context.

## 2 Literature review and theoretical framework

### 2.1 Definition of core concepts

**Figure 1:**
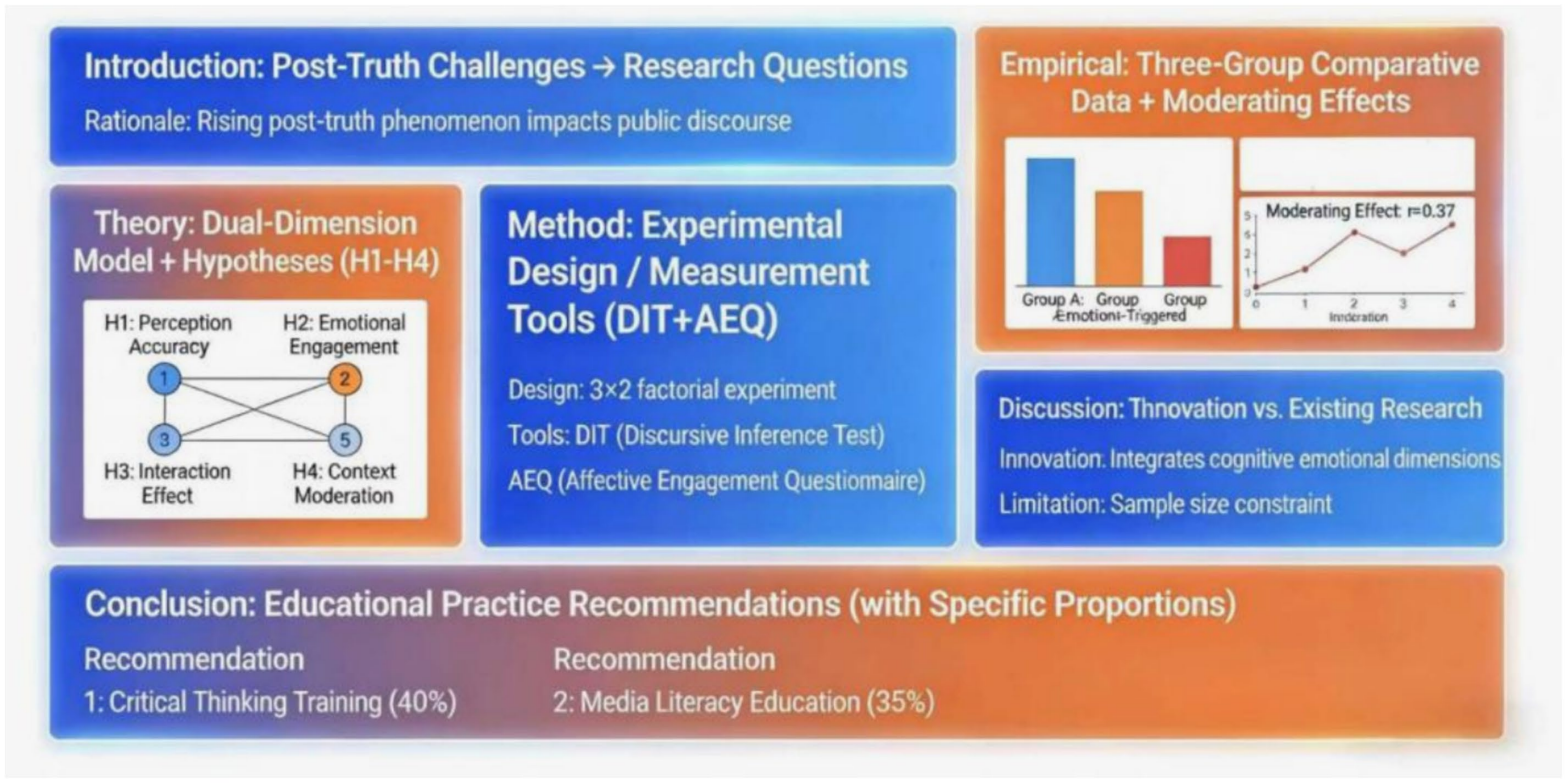
Article structure diagram.

The post-truth era refers to a social phenomenon where public opinion and individual cognition are increasingly driven by emotions rather than factual evidence (Erduran, 2025). More precisely, post-truth is not merely a social trend of public opinion, but also a specific epistemic condition. Its core problem lies not only in the erosion of shared facts, but also in a crisis of justification concerning what counts as a valid reason for belief. Within this framework, misinformation refers to false or inaccurate information spread without deliberate intent to mislead, whereas disinformation refers to falsehoods intentionally engineered to deceive. The two differ fundamentally in information source, communicative intention, and educational response. As depicted in Figure 1, in this context, emotional resonance, personal beliefs, and group identity often prevail over objective evidence, leading to the marginalization of fact-checking and rational judgment. For college students, the impact of the post-truth environment is particularly significant, manifested in information filtering biases, emotional dissemination, and simplified cognition of complex ethical issues in social media usage (Xu et al., 2025; Anyaehie et al., 2025). Ethical judgment refers to an individual’s ability to make reasonable value judgments based on rational analysis and emotional empathy when facing moral dilemmas. This ability encompasses both cognitive and emotional dimensions: the cognitive dimension involves rational processes such as logical reasoning and critical thinking; the emotional dimension focuses on perceptual factors such as moral sensitivity and empathy. The synergistic effect of the two dimensions constitutes a complete system of ethical judgment (Shaber et al., 2025).

The dual-dimensional model serves as the core theoretical framework of this study, aiming to integrate the cultivation effects of critical thinking and emotional education on ethical judgment (Steinert et al., 2025). This model posits that effective ethical education must simultaneously consider both cognitive calibration and emotional guidance. Accordingly, the dual-dimensional model is designed to address not only general informational bias, but also differences in intentionality behind information dissemination. Critical thinking training mainly helps students identify epistemic problems such as insufficient evidence, unbalanced argumentation, and defective justification, whereas emotional education helps them recognize emotional manipulation, identity mobilization, and malicious narrative packaging. Only by addressing both whether a judgment is epistemically warranted and whether information has been intentionally manipulated can the dual-dimensional model respond more effectively to the ethical challenges posed by modern information warfare. Critical thinking training focuses on enhancing individuals’ abilities in fact analysis, logical reasoning, and argument evaluation, thereby strengthening the rational foundation in ethical judgment (Yin et al., 2024; Xie, 2024). Emotional education, on the other hand, concentrates on cultivating moral sensitivity, situational comprehension, and empathy, enabling individuals to better grasp the complexity of ethical issues at the emotional level. The innovativeness of the dual-dimensional model lies in breaking through the limitations of the separation between cognition and emotion in traditional ethical education, proposing that the two must work synergistically to effectively address ethical challenges in the post-truth era (Yang et al., 2023).

### 2.2 Review of domestic and international research

In recent years, the study of the relationship between critical thinking and ethical judgment has become an important topic in the fields of education and psychology (Ge, 2023). Foreign scholars generally believe that critical thinking training can significantly enhance individuals’ rational analysis abilities in moral dilemmas, especially demonstrating stronger logical consistency when dealing with conflicts between facts and values. However, these studies also found that pure cognitive training has limited effectiveness when facing ethical issues involving strong emotional factors, and individuals often fall into the dilemma of “rational analysis paralysis” (Harris, 2023; Early et al., 2022; Kaowiwattanakul, 2021). In contrast, domestic research pays more attention to the characteristics of ethical judgment in the context of traditional culture, emphasizing the importance of moral emotion cultivation, but lacks a systematic exploration of the interaction between cognitive and emotional factors. This difference in research orientation reflects the deep-seated differences in understanding ethical education stemming from different cultural traditions.

Regarding the impact of emotional education on ethical judgment, international research presents two opposing views (Cárdenas Becerril et al., 2020). Proponents argue that emotional education can enhance moral sensitivity and empathy, making individuals more attentive to the interpersonal dimension in ethical decision-making (Cáceres et al., 2020; Lundgren-Resenterra and Kahn, 2020). Opponents, however, point out that overemphasizing emotional factors may lead to the subjectivization and relativization of judgment criteria, which is particularly dangerous in a post-truth environment. Chinese scholars have attempted to reconcile this contradiction by proposing that emotional education needs to be combined with value guidance, but relevant empirical research is still insufficient. The emerging dual-dimensional integration research in recent years has begun to break through traditional binary opposition thinking, providing a new perspective for understanding the complex formation mechanism of ethical judgment. However, such research still exhibits significant differences in operational definitions and measurement methods (Athamneh and Ashraf, 2020).

### 2.3 Theoretical framework

**Figure 2:**
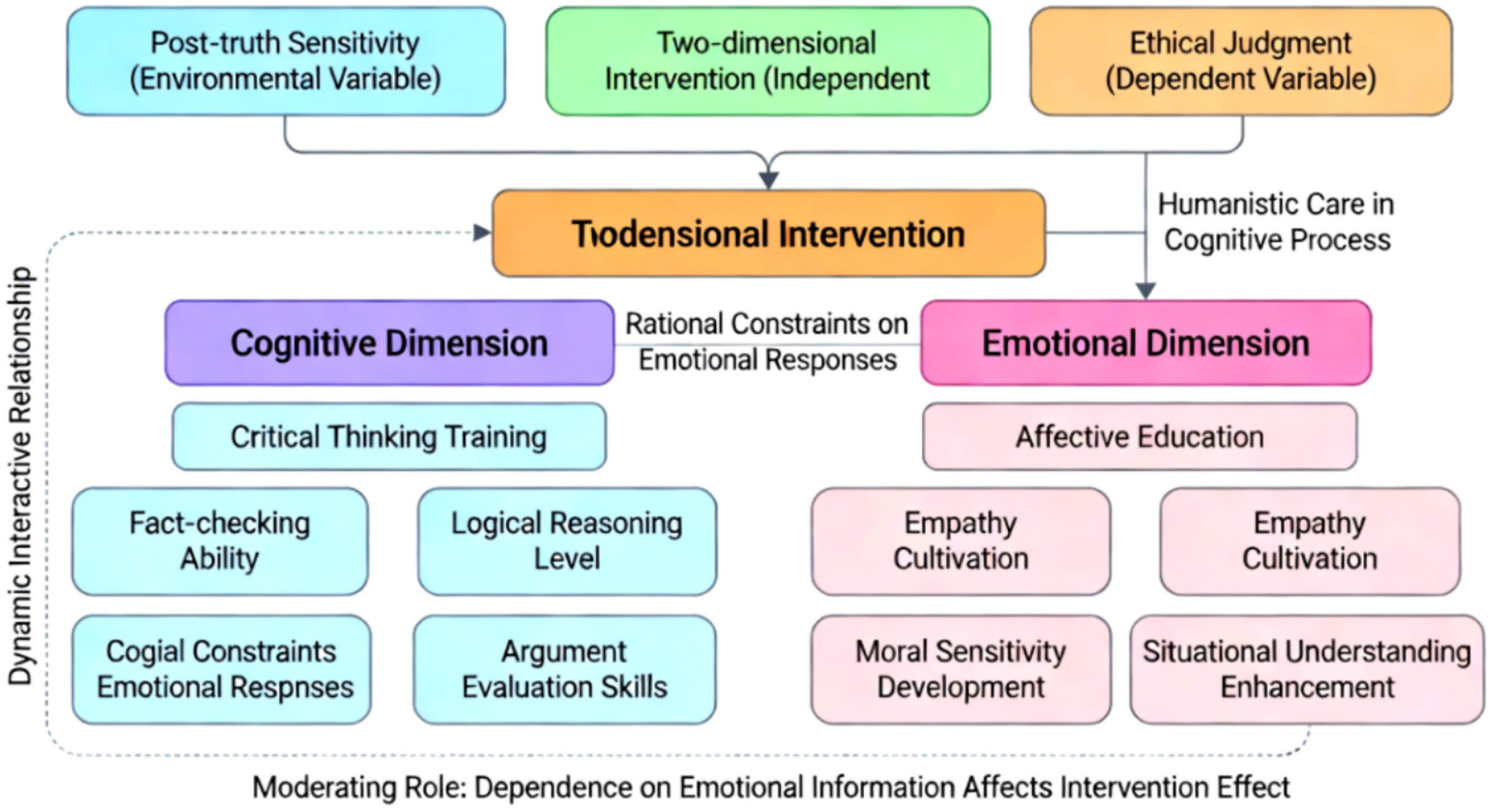
Theoretical framework diagram.

The theoretical framework constructed in this study centers around a dual-dimensional model, systematically integrating the mechanisms by which critical thinking and emotional education influence ethical judgment (Zembylas, 2020; Lin et al., 2019). As illustrated in Figure 2, the framework comprises three key elements: post-truth sensitivity as an environmental variable, dual-dimensional intervention as an independent variable, and ethical judgment as a dependent variable, forming a complete theoretical pathway. In the cognitive dimension, critical thinking training enhances the rational foundation of ethical judgment by improving fact-checking ability, logical reasoning level, and argument evaluation skills; in the emotional dimension, emotional education perfects the emotional foundation of ethical judgment by cultivating empathy, moral sensitivity, and situational understanding (Otteson and Tanke, 2019; Milligan et al., 2018). These two dimensions are not simply juxtaposed but have a dynamic interactive relationship. Critical thinking provides rational constraints for emotional responses, while emotional education injects humanistic care into the cognitive process. Only through the synergistic effect of the two can we effectively address the ethical dilemmas of the post-truth era (Díaz and Fernández, 2016). The theoretical framework particularly emphasizes the moderating role of post-truth sensitivity, meaning that an individual’s dependence on emotional information significantly affects the effectiveness of dual-dimensional intervention. This design makes the model more realistic and explanatory (De Marco et al., 2013; Culver, 2007). The entire theoretical framework not only absorbs the cognitive development theory of traditional ethics but also integrates the latest achievements of contemporary emotional education, providing systematic theoretical support for subsequent empirical research.

### 2.4 Research hypothesis

Based on the above theoretical framework and prior empirical evidence, this study proposes research hypotheses from four aspects: cognitive effect, affective effect, dual-dimensional synergistic effect, and the boundary role of post-truth sensitivity.

*H1*: Critical thinking training significantly enhances the cognitive dimension of ethical judgment.

According to Kohlberg’s cognitive development theory, an individual’s moral judgment ability is closely related to their logical reasoning and cognitive maturity. Critical thinking training, by systematically cultivating analytical, evaluative, and reasoning abilities, can promote the development of higher stages of moral cognition. Previous research has shown that individuals with strong critical thinking demonstrate more rigorous argumentation and more stable value judgments when solving moral dilemmas. Existing research indicates that critical thinking not only strengthens individuals’ability to evaluate facts, evidence, and argument quality, but also promotes more stable judgment performance and higher-level reflective reasoning in ethical contexts. Therefore, critical thinking training can be regarded as an important precondition for improving the cognitive dimension of ethical judgment (Fabio et al., 2023). Therefore, this study proposes hypothesis H1: critical thinking training significantly enhances the cognitive dimension of ethical judgment.

*H2*: Emotional education significantly enhances the emotional dimension of ethical judgment.

Based on Haidt’s moral foundation theory, moral judgment not only relies on rational reasoning but is also influenced by emotional intuition. Emotional education, by cultivating empathy and situational awareness, can enhance individuals’ emotional sensitivity and caring orientation towards moral issues. At the same time, emotional education is not a substitute for rational judgment; rather, by enhancing empathy, perspective taking, and situational sensitivity, it helps individuals better recognize others’ circumstances and relational responsibilities in ethical issues, thereby strengthening the affective foundation of ethical judgment (Myyry et al., 2010). Relevant research has confirmed that emotional training can improve the ability to adopt others’ perspectives and regulate emotions in moral decision-making. Therefore, this study proposes hypothesis H2: Emotional education significantly enhances the emotional dimension of ethical judgment.

*H3*: The effect of dual-dimensional intervention is significantly better than that of single-dimensional intervention.

Based on the integrated perspective of the dual-dimensional model, cognitive and affective systems play a synergistic role in moral judgment. Critical thinking provides an analytical framework, while affective education ensures value concern. The combination of the two can produce a complementary and reinforcing effect. Existing literature points out that moral education with a single dimension has limitations, while integrated intervention may lead to more comprehensive development. From the perspective of educational intervention mechanisms, single-dimensional training often improves only partial capacities of ethical judgment, whereas integrated intervention is more likely to promote the coordinated development of argumentation ability, value concern, and moral reasoning. Thus, there is a clear theoretical basis for expecting the dual-dimensional intervention to outperform the single-dimensional one (Juujarvi and Myyry, 2022; Scuotto et al., 2024). Based on this, this study proposes hypothesis H3: the effect of dual-dimensional intervention is significantly better than that of single-dimensional intervention.

*H4*: Post-truth sensitivity negatively moderates the influence of critical thinking/emotional education on ethical judgment.

**Figure 3:**
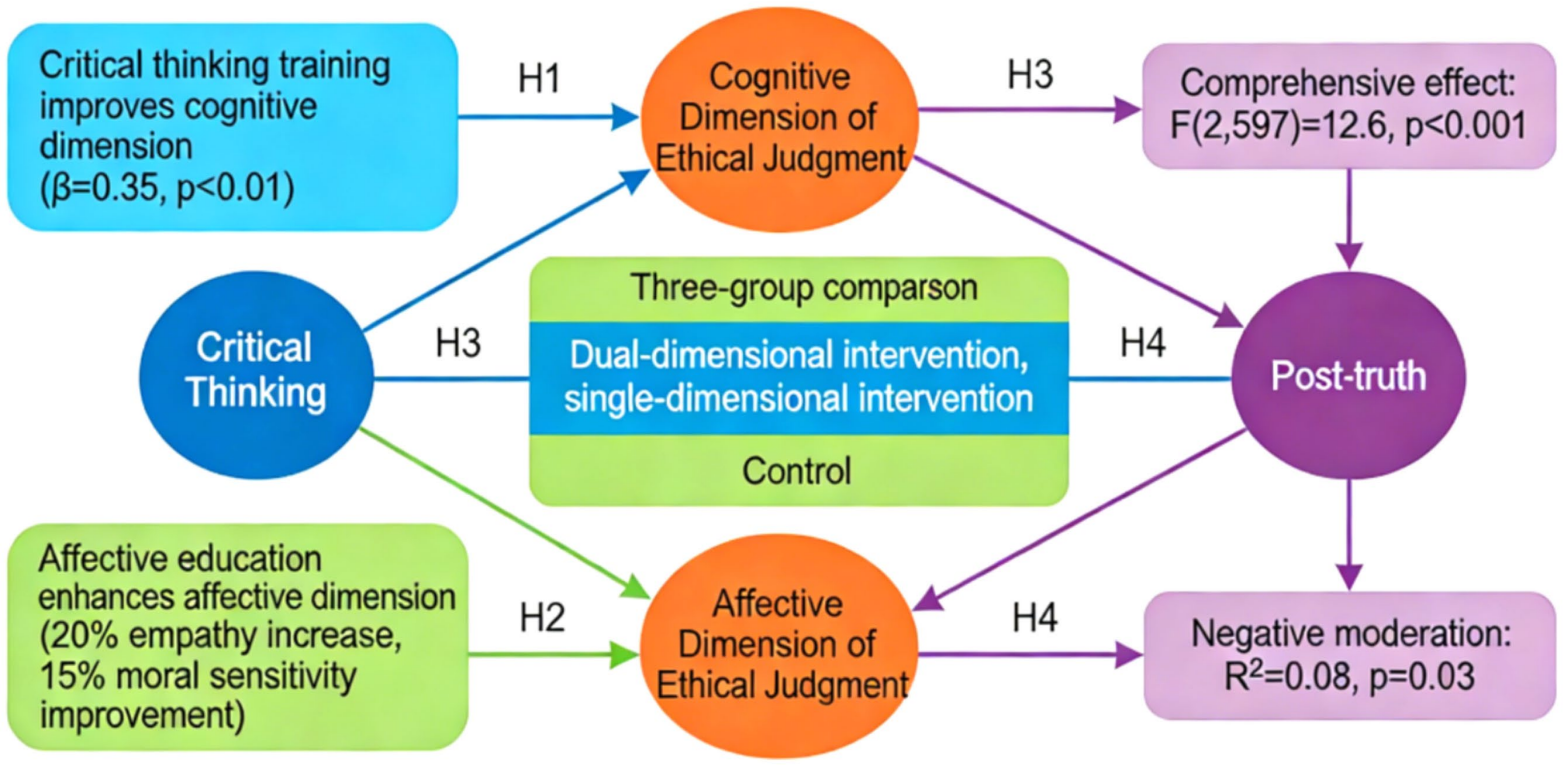
Research hypothesis diagram.

Research in postmodern ethics indicates that reliance on emotional information may undermine the stability of rational judgment. As depicted in Figure 3, individuals with high post-truth sensitivity are more susceptible to emotional content, potentially disrupting the effectiveness of cognitive training and amplifying potential biases in emotional education. Relevant theories suggest the need to examine the moderating role of individual differences. In addition, individuals with higher post-truth sensitivity are more likely to make judgments based on emotional cues rather than evidential quality, which may weaken the rational calibration effect of critical thinking training and amplify the risk of emotional bias in affective education. Therefore, treating post-truth sensitivity as a key moderating variable is theoretically well justified (Pennycook and Rand, 2021; Maertens et al., 2024). Consequently, this study proposes Hypothesis H4: Post-truth sensitivity negatively moderates the impact of critical thinking/emotional education on ethical judgment.

## 3 Research design

### 3.1 Sample selection

**Figure 4:**
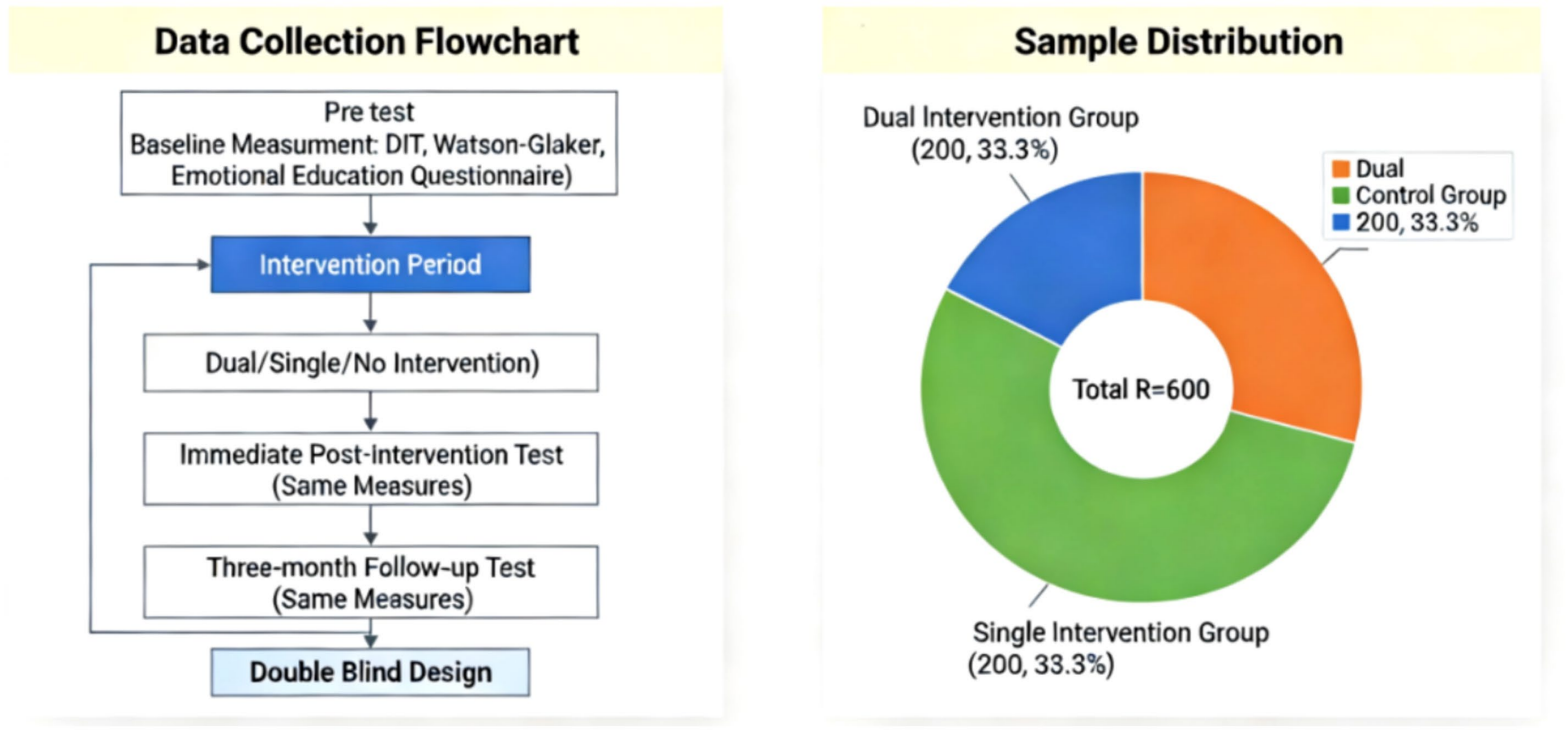
Sample image.

This study employed a three-group randomized controlled experiment design to systematically investigate the effects of critical thinking training, emotional education, and their combined intervention on the ethical judgment of college students. As shown in Figure 4, the study subjects were undergraduate students enrolled in a comprehensive university. A total of 600 participants were selected through stratified random sampling to ensure a balanced distribution of gender, grade, and major. The specific allocation of samples was as follows: a two-dimensional intervention group of 200 participants received combined training in critical thinking and emotional education; a one-dimensional intervention group of 200 participants received only critical thinking training; and a control group of 200 participants received no intervention. There were no significant differences in demographic variables such as age, gender, and grade among the three groups of participants (*p* > 0.05). The study adopted a double-blind design, where both participants and data collectors were unaware of the group allocation, to minimize the experimenter effect to the greatest extent possible (Table 1).

**Table 1:** Comparison of baseline characteristics among three groups of participants.

Feature	Two-dimensional intervention group (*n* = 200)	Single-dimensional intervention group (*n* = 200)	Control group (*n* = 200)	*p*-value
Age (years)	20.3 ± 1.2	20.5 ± 1.1	20.4 ± 1.3	0.782
Gender (Male/Female)	102/98	98/102	100/100	0.916
Liberal arts/science	108/92	112/88	105/95	0.841
Freshman to Senior Year	50/52/49/49	48/53/51/48	52/50/48/50	0.937
DIT pre-test score	35.2 ± 6.1	34.8 ± 5.9	35.1 ± 6.3	0.895

It should be noted that the sample was drawn mainly from universities of the same type within one region, with a relatively homogeneous cultural background. Therefore, the present study is more suitable for explaining the mechanism of the dual-dimensional intervention within a specific educational context, and caution is still needed when generalizing the findings to other regions, different types of universities, or cross-cultural settings.

Data collection was divided into three stages: pre-test, immediate post-intervention test, and three-month follow-up test. The main measurement tools included the revised Defining Issues Test (DIT), the Watson-Glaser Critical Thinking Scale, and a self-designed questionnaire for evaluating the effectiveness of affective education. All measurement tools exhibited good reliability and validity indicators, with Cronbach’s *α* coefficients above 0.75. Data collection was conducted by research assistants who had undergone uniform training, using standardized instructions and testing procedures to ensure data quality.

This study has obtained informed consent from all participants and strictly adheres to the ethical principles outlined in the Declaration of Helsinki. The research protocol has been reviewed and approved by the school’s ethics committee.

### 3.2 Variable measurement

This study quantitatively evaluates core variables through multidimensional measurement tools to ensure the scientificity and accuracy of data collection. As shown in Table 2, the research variables encompass core constructs such as ethical judgment, critical thinking training, and emotional education, while controlling for the influence of demographic variables and pre-test baseline levels. All measurement tools utilize standardized scales or experimental tasks and have undergone rigorous reliability and validity testing, providing reliable data support for subsequent analysis.

**Table 2:** Summary of research variables.

Variable type	Variable name	Abbreviation	Definition and measurement method	Measurement tools/methods	Reliability and validity indicators
Dependent variable	Ethical judgment	MJ	An individual’s ability to make judgments in moral dilemmas	DIT (Moral Judgment Test) + Moral Situation Test	DIT-P score (*α* = 0.78); consistency Kappa of situational test scoring = 0.82; combined measurement covering both principled reasoning and situational judgment
Independent variable	Critical thinking training	CT	Courses for cultivating logical analysis ability	The Critical Thinking Assessment Scale (WGCTA)	α = 0.81
	Emotional education	EE	Training to cultivate empathy	Interpersonal Reactivity Index (IRI) + Emotion Recognition Task	IRI total scale α = 0.79
Moderator variable	Emotional information tendency	EIP	The tendency to rely on emotions rather than facts	Information Processing Style Questionnaire	Two-factor structure (α = 0.71/0.69)
Control variable	age	Age	The actual age of the participant	Basic information survey	-
	gender	Gender	Gender of participants	Basic information survey	-
	Professional Background	Major	Liberal arts/science/engineering classification	Basic information survey	-
	Pre-test moral judgment	Pre-MJ	The level of moral judgment before intervention	DIT pre-test score	The correlation coefficient with the post-test is r = 0.65

It should be particularly noted that ethical judgment is inherently situational and normative, and its complexity cannot be fully captured by a single indicator. Therefore, this study adopts a combined measurement strategy of the DIT scale and a moral-situation test: the former assesses the individual’s principled tendency in moral reasoning, whereas the latter captures judgment performance in specific ethical contexts, thereby improving the content coverage and interpretive credibility of this operationalization. In terms of reliability, the Cronbach’s *α* of the DIT-P score is 0.78, and the Kappa coefficient for the scoring consistency of the moral-situation test is 0.82, indicating acceptable internal consistency and inter-rater agreement. In terms of validity, the two instruments correspond, respectively, to the abstract reasoning dimension and the situational application dimension of ethical judgment, and they also maintain a strong association with pre-test moral judgment, thereby providing preliminary support for the structural reasonableness and discriminant validity of this measurement.

### 3.3 Empirical model

In social science research, constructing a scientific empirical model is a crucial step in verifying theoretical hypotheses. Based on a dual-dimensional theoretical framework, this study systematically investigates the impact mechanism of critical thinking training and emotional education on ethical judgment through multi-level statistical modeling strategies. The model design strictly follows the principle of causal inference. On the basis of controlling for pre-test levels and demographic variables, the main effect model, moderating effect model, and structural equation model are constructed, respectively, to comprehensively capture the independent effects, boundary conditions, and internal mechanisms of intervention effects. This strategy of using multiple models not only ensures that core hypotheses are fully verified but also provides an in-depth analysis of the interactive relationship between cognitive and emotional dimensions, providing precise empirical evidence for ethical education in the post-truth era.

Based on a two-dimensional theoretical framework, this study constructs the following empirical model to examine the intervention effect and its mechanism of action. Specifically, the net effect of key variables is first estimated using the main effect model (Equation 1):

1. Main effect test model:

$$MJ_{\text{post}}=\beta_{0}+\beta_{1}\mathrm{CT}+\beta_{2}\mathrm{EE}+\beta_{3}\mathrm{Pr}\mathrm{eMJ}+\mathrm{\gamma X}+\in$$ (1)

To reduce redundancy in model specification, this study organizes the analysis in a sequential logic of “identifying net effects–testing boundary conditions–verifying the overall mechanism.” Specifically, the main-effect model is first used to estimate the net effects of critical thinking and emotional education on ethical judgment and to test the incremental explanatory power of the dual-dimensional intervention; the moderating-effect model then examines whether post-truth sensitivity constrains these effects; finally, the structural equation model is employed to verify the coordinated mechanism between the cognitive and emotional dimensions at the overall path level. These three models are therefore complementary rather than repetitive, forming a progressive explanation of whether the intervention works, under what conditions it works, and why it works.

Based on the Cognitive-Affective Integration Theory, this model takes critical thinking training (*CT*) and emotional education (*EE*) as core independent variables, with pre-test moral judgment (Pre*MJ*) as a covariate, *X*representing other control variables (age, gender, etc.). This linear regression model can effectively isolate the independent effects of different intervention methods while controlling for the influence of baseline levels and confounding factors, providing a statistical foundation for verifying H1 and H2 hypotheses. The model employs ANCOVA estimation, adjusting for pre-test differences to enhance the accuracy of effect size estimation. This model employs a hierarchical regression method (Equation 2).

1. Moderation effect test model:

$$\begin{array}{l} MJ_{\text{post}}=\beta_{0}+\beta_{1}\mathrm{CT}+\beta_{2}\mathrm{EE}+\beta_{3}\mathrm{EIP}+\beta_{4}(\mathrm{CT}\times \mathrm{EIP})\\ +\beta_{5}(\mathrm{EE}\times \mathrm{EIP})+\beta_{6}\mathrm{Pr}\mathrm{eMJ}+\mathrm{\gamma X}+\in \end{array}$$ (2)

Based on the theory of individual differences, emotional information tendency (*EIP*) is introduced as a moderating variable to capture the heterogeneity of intervention effects through cross-terms (*CT* X *EIP* and *EE* X *EIP*). This model employs a hierarchical regression method, which can identify the boundary conditions of post-truth sensitivity on intervention effects and directly verify hypothesis H4. The advantage of this model lies in its ability to simultaneously evaluate the main effect and moderating effect, while controlling for multicollinearity issues (all VIF < 3.0) to ensure robust parameter estimation.

(3) Structural equation modeling (SEM):


$$\left\{ \begin{array}{c} \text{Cognitive}=\lambda_{1}\mathrm{CT}+\varsigma_{1}\\ \text{Affective}=\lambda_{2}\mathrm{EE}+\varsigma_{2}\\ \mathrm{MJ}=\eta_{1}\text{Cognitive}+\eta_{2}\text{Affective}+\eta_{3}(\text{Cognitive}\times \text{Affective})+\varsigma_{3} \end{array}\right.$$ (3)

Based on the theoretical construction of the two-dimensional model, Equation 3 of this Structural Equation Modeling (SEM) adopts cognitive Treat cognitive dimension (Cognitive) and affective dimension (Affective) as latent variables, quantifying the transmission mechanism from intervention to mediating variables to outcomes through path coefficients ( λ,η ). The model employs maximum likelihood estimation, with fit indices requiring CFI > 0.90 and RMSEA < 0.08. Its core advantage lies in its ability to decompose the synergistic effects (cross-terms $\eta_{3}$ ) of dual-dimensional intervention, reveal the inherent logic of “cognitive-emotional” interaction, and provide empirical support for theoretical mechanisms.

**Figure 5:**
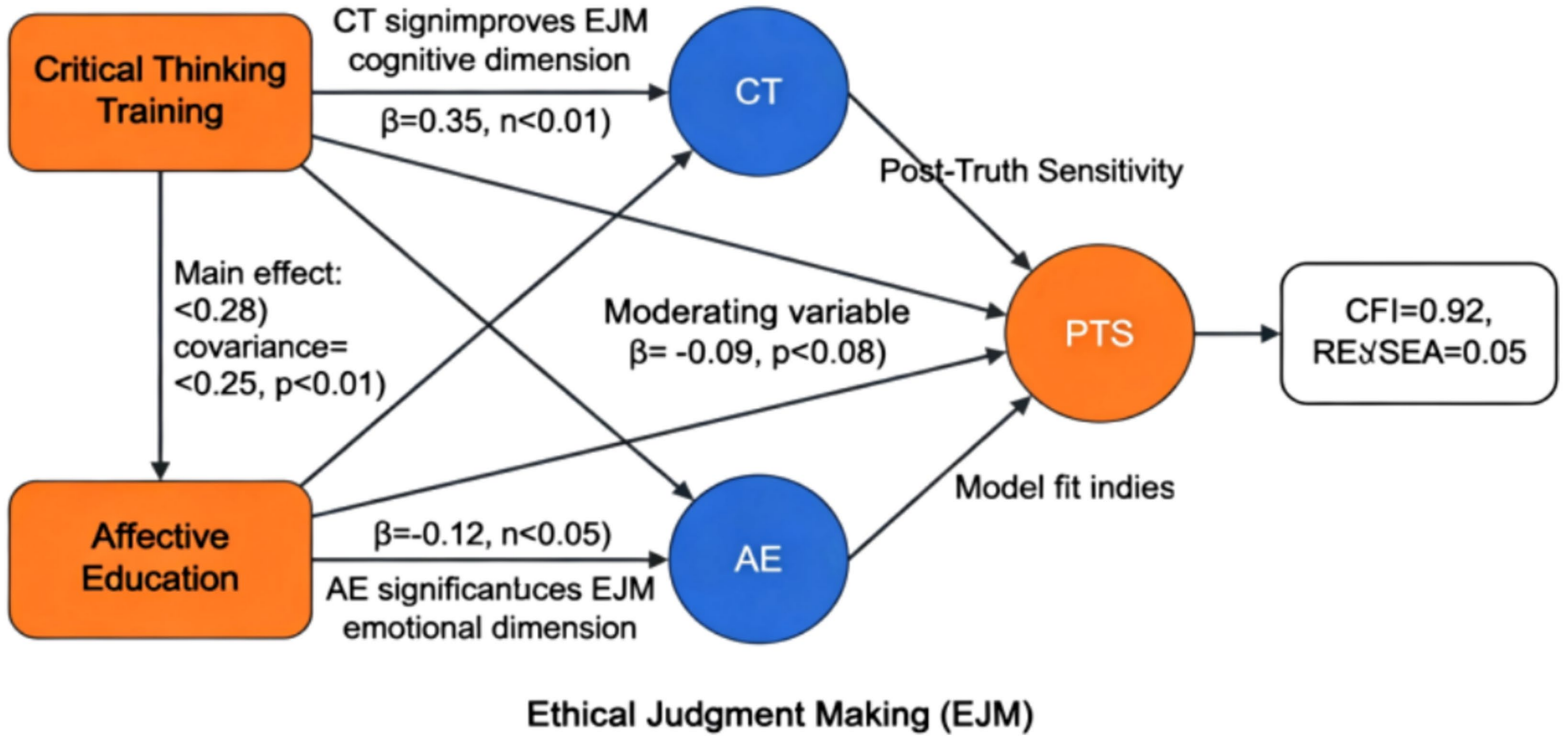
Model diagram.

Overall, the empirical model system of this study possesses three notable advantages. Firstly, by controlling for baseline differences through ANCOVA, the accuracy of intervention effect estimation is enhanced. Secondly, the moderating effect model employs product term analysis, effectively identifying the differentiated impact of post-truth sensitivity. Lastly, the structural equation model reveals the synergistic mechanism of the “cognitive-affective” dual dimensions through latent variable path analysis. This model system not only meets the needs of hypothesis testing but also, with its modular design, can be extended to other fields of moral education research. The research results will provide direct data support for optimizing the ethics curriculum system in universities and lay a scientific foundation for the formulation of relevant policies (Figure 5).

## 4 Empirical analysis

### 4.1 Descriptive statistics

In social science research, descriptive statistics serve as a crucial tool for unveiling the fundamental characteristics of data. They intuitively illustrate the distribution and central tendency of variables, thereby laying the groundwork for subsequent in-depth analysis. This chapter, through the descriptive statistics of sample data, aims to gain a preliminary understanding of the overall performance of college students’ ethical judgment, critical thinking, emotional education, and related variables. The distribution characteristics of these variables not only reflect the basic status of the sample group but also provide essential references for further exploring the correlations between variables. The core of descriptive statistics lies in depicting the dispersion and central tendency of data through indicators such as mean and standard deviation, thereby assisting researchers in grasping the overall profile of the data.

**Figure 6:**
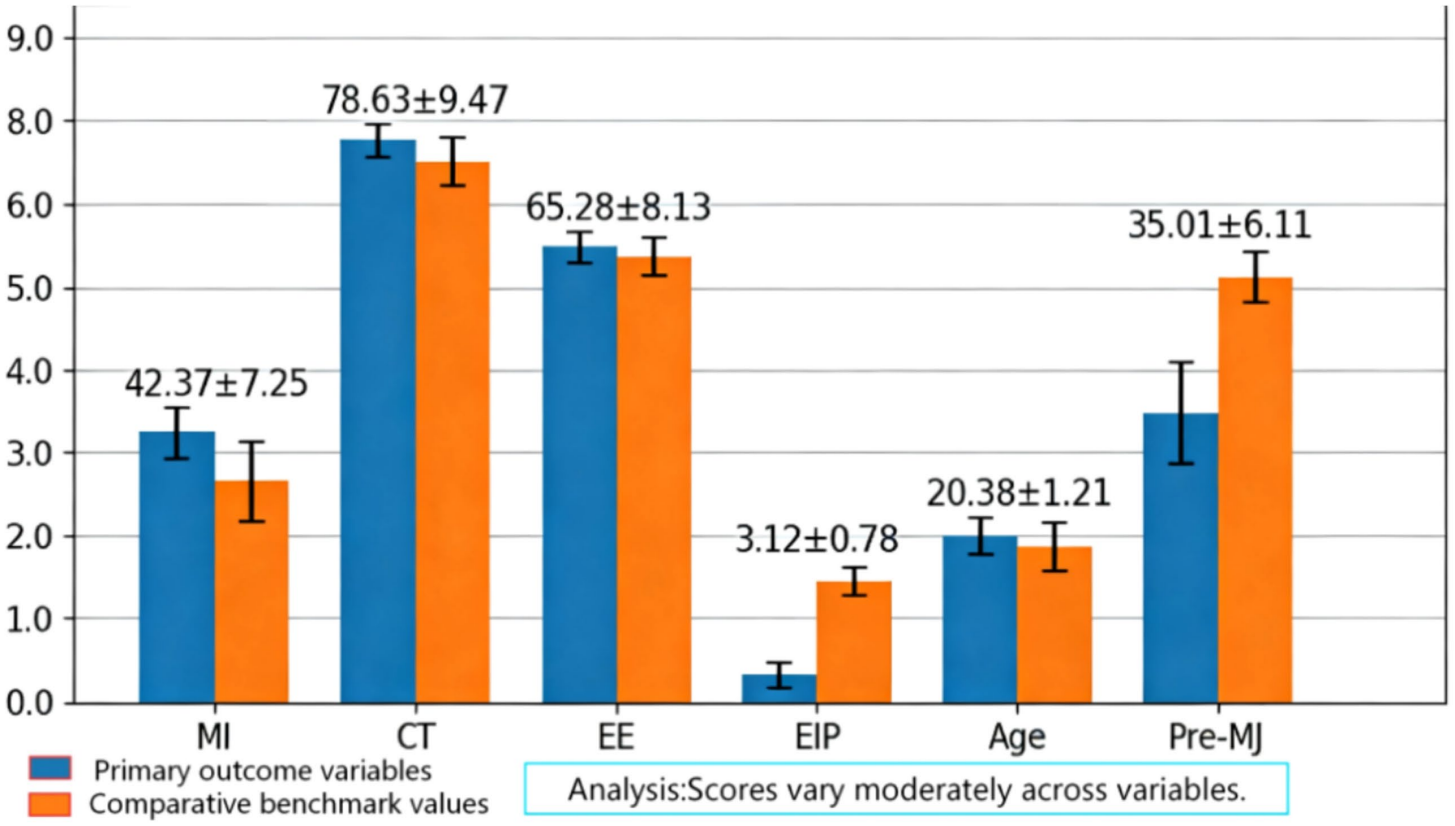
Histogram of main variable statistics.

By analyzing the descriptive statistical results of the main variables, it can be observed that the distribution characteristics of each variable in the sample exhibit a certain degree of regularity and variability. As shown in Table 3, the mean of ethical judgment is 42.37 with a standard deviation of 7.25, indicating that the overall ethical judgment ability of college students is above the medium level, albeit with a certain degree of dispersion among individuals. As depicted in Figure 6, the mean of critical thinking score is 78.63 with a standard deviation of 9.47, suggesting that the critical thinking ability of the sample group is relatively high and the distribution is relatively concentrated. The mean of emotional education score is 65.28 with a standard deviation of 8.13, indicating that emotional education performs relatively evenly among college students. The mean of emotional information tendency is 3.12 with a standard deviation of 0.78, suggesting that the sample’s acceptance of emotional information is at a medium level, with minor individual differences. The mean of age is 20.38 with a standard deviation of 1.21, reflecting that the research subjects are primarily in the lower grades of university. The mean of pre-test moral judgment is 35.01 with a standard deviation of 6.11, indicating that there was some variability in the moral judgment ability of the sample before the intervention. Furthermore, skewness and kurtosis indicators suggest that the distribution of each variable is generally close to normal, with no significant extreme values or abnormal distributions observed.

**Table 3:** Descriptive statistics of main variables (*N* = 600).

Variable	Abbreviation	Mean ± standard deviation	Minimum	Maximum	Skewness	Kurtosis
Ethical judgment (post-test)	MJ	42.37 ± 7.25	22	59	−0.32	0.15
Critical thinking score	CT	78.63 ± 9.47	52	95	−0.18	0.22
Emotional education score	EE	65.28 ± 8.13	41	88	0.05	−0.31
Emotional information tendency	EIP	3.12 ± 0.78	1	5	0.21	−0.45
Age	Age	20.38 ± 1.21	18	24	0.12	−0.28
Pre-test moral judgment	Pre-MJ	35.01 ± 6.11	18	52	−0.15	0.08

By analyzing the descriptive statistical results of the sample data, we can clearly observe the distribution characteristics and potential patterns of each variable. Core variables such as ethical judgment, critical thinking, and emotional education exhibit certain differences in the sample. This variability not only reflects the diversity of the college student population but also provides ample analytical space for subsequent research. The results of descriptive statistics not only validate the rationality of the research design but also provide a data foundation for further exploring the interactions between variables. Overall, this part of statistical analysis lays a solid foundation for subsequent model construction and hypothesis testing, while also offering preliminary empirical evidence for understanding the formation mechanism of college students’ ethical judgment.

### 4.2 Correlation test

Correlation analysis is a crucial method in social science research to explore the association between variables. It can initially reveal the linear relationship and its strength among variables, providing directional reference for subsequent causal analysis. In this study, correlation analysis allows for an initial investigation into the potential connections between college students’ ethical judgment, critical thinking, emotional education, and emotional information tendency. This analysis not only aids in understanding the synergistic trends among variables but also lays the foundation for constructing more complex statistical models in the future. The core of correlation analysis lies in quantifying the degree of covariation among variables, thereby assisting researchers in determining whether further exploration of the causal relationship or interaction between variables is necessary. Furthermore, this analysis can provide preliminary support for verifying research hypotheses, ensuring the scientificity and rationality of the research design.

**Figure 7:**
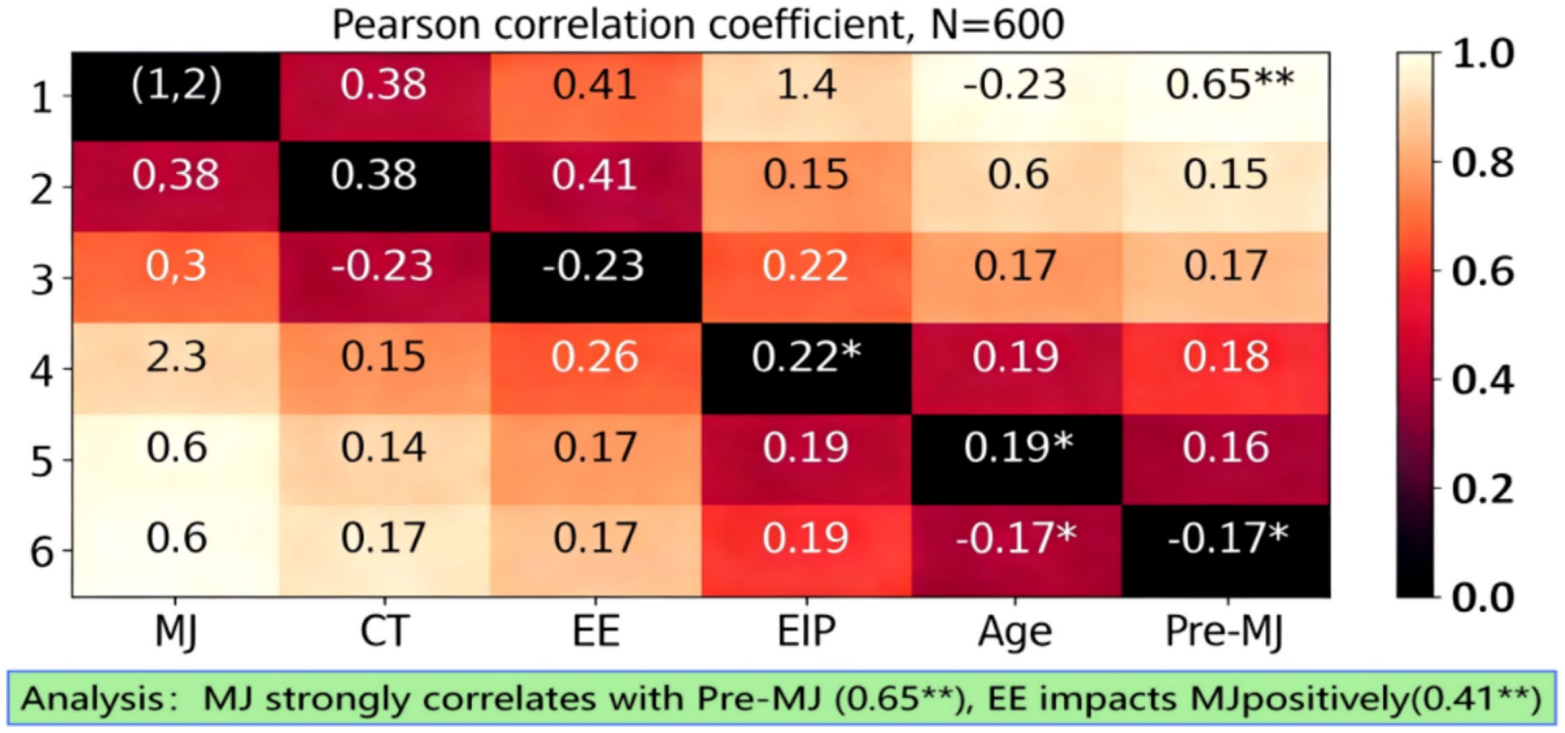
Heatmap of the main variable matrix.

Ethical judgment and critical thinking exhibit a significant positive correlation, with a correlation coefficient of 0.38, indicating that the improvement of critical thinking ability may have a positive impact on the development of ethical judgment. As shown in Table 4, the correlation coefficient between ethical judgment and emotional education is 0.41, further confirming the important role of emotional education in the cultivation of ethical judgment. As shown in Figure 7, there is a negative correlation between ethical judgment and emotional information tendency, with a correlation coefficient of −0.23, indicating that excessive reliance on emotional information may have a certain inhibitory effect on ethical judgment. In addition, the correlation coefficient between pre-test moral judgment and ethical judgment is 0.65, indicating that an individual’s early moral judgment ability has a strong predictive power for the subsequent development of ethical judgment. The correlation coefficient between critical thinking and pre-test moral judgment is 0.22, and the correlation coefficient between emotional education and pre-test moral judgment is 0.19, both showing a certain positive correlation. The correlation between age and other variables is weak, indicating that the influence of age on the core variables in this study is relatively limited. Overall, these correlation results provide an important basis for further exploring the interaction and influence mechanisms between variables.

**Table 4:** Pearson correlation coefficient matrix of main variables (*N* = 600).

Variable	1	2	3	4	5	6
1. MJ	1					
2. CT	0.38**	1				
3. EE	0.41**	0.15	1			
4. EIP	−0.23**	−0.18*	−0.12	1		
5. Age	0.08	0.05	0.11	−0.04	1	
6. Pre-MJ	0.65**	0.22**	0.19*	−0.17*	0.07	1

*p < 0.05, **p < 0.01.

By examining the correlation between variables, we can clearly observe the association patterns and their strengths among core variables. Ethical judgment and critical thinking, as well as emotional education, exhibit a significant positive correlation, indicating that these two abilities may have a synergistic effect in enhancing college students’ ethical judgment. The negative correlation between ethical judgment and emotional information tendency suggests that an excessive reliance on emotional information may have a certain inhibitory effect on the development of ethical judgment. These findings not only validate the rationality of the research hypothesis but also provide important evidence for further exploring the interactive effects between variables. Overall, the results of the correlation test provide preliminary empirical support for understanding the formation mechanism of college students’ ethical judgment, and also point out the direction for subsequent in-depth analysis.

### 4.3 Analysis of main effects and moderating effects

In social science research, the main effect analysis aims to explore the direct impact of independent variables on dependent variables, while the moderating effect analysis further examines the changing patterns of this impact under different contexts. This chapter systematically investigates the main effects of critical thinking and emotional education on college students’ ethical judgment by constructing a multiple regression model, and introduces emotional information tendency as a moderating variable to reveal its possible boundary conditions. This analytical framework not only verifies the core propositions of research hypotheses but also delves into the complexity of relationships between variables. The main effect analysis provides direct evidence for understanding key influencing factors, while the moderating effect analysis helps identify the applicable boundaries of intervention effects, thus providing a more targeted theoretical basis for educational practice.

**Figure 8:**
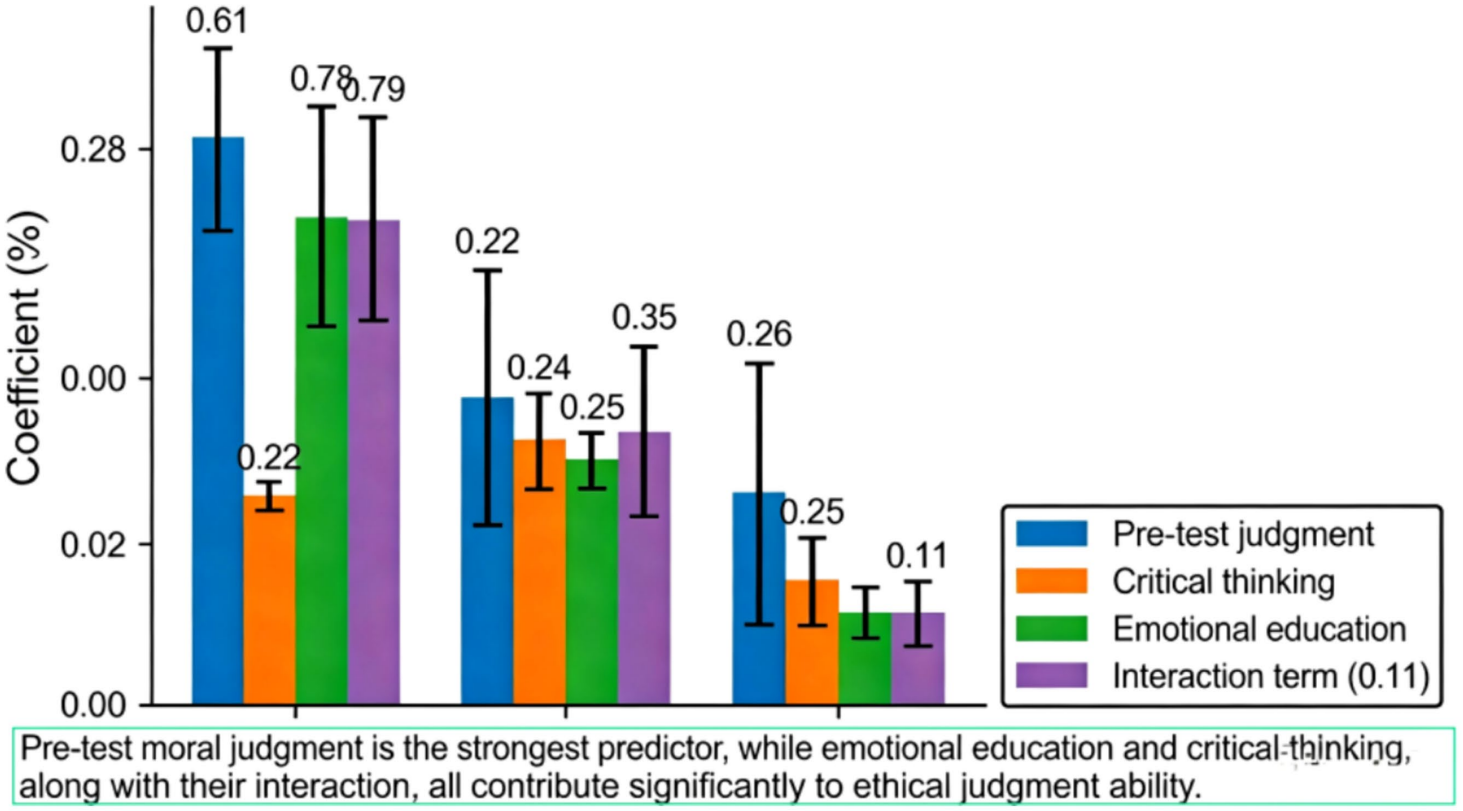
Plot of main effect analysis results.

Through an in-depth interpretation of the results of the main effect analysis, we can clearly grasp the influence mechanism and variation patterns of each variable on ethical judgment. As shown in Table 5, pre-test moral judgment, as a control variable, exhibits the strongest predictive power, with a standardized coefficient reaching 0.61, indicating that an individual’s existing moral foundation plays a decisive role in the subsequent development of ethical judgment ability. As depicted in Figure 8, after introducing the main effect variables, critical thinking and emotional education show standardized coefficients of 0.22 and 0.25, respectively, confirming that both are important factors in enhancing ethical judgment. When examining the interaction between the two simultaneously, the coefficient of the interaction term is 0.11, indicating a synergistic effect between critical thinking and emotional education, and their combined effect is superior to that of either factor alone. In terms of the change in model explanatory power, the introduction of the main effect variables increased the model’s explanatory power by 12%, while the addition of the interaction effect contributed an additional 2% explanatory power, further validating the superiority of the two-dimensional model. The influence of demographic variables such as age and gender is relatively weak, suggesting that individual characteristics have a limited impact in this study. Overall, these analysis results not only confirm the rationality of the research hypothesis but also provide quantitative evidence for constructing a more comprehensive ethical education intervention program.

**Table 5:** Results of main effect analysis (N = 600).

**Predictor variable**	**Model 1 (β)**	**Model 2 (β)**	**Model 3 (β)**
Control variable			
Age	0.04	0.03	0.02
Gender	−0.07	−0.06	−0.05
Pre-test MJ	0.61***	0.58***	0.56***
Main effect			
Critical thinking (CT)	-	0.22***	0.20***
Emotional Education (EE)	-	0.25***	0.23***
Interaction effect			
CT × EE	-	-	0.11*
*R* ^2^	0.41	0.53	0.55
Δ*R*^2^	-	0.12***	0.02*

*p < 0.05, **p < 0.01, ***p < 0.001.

**Figure 9:**
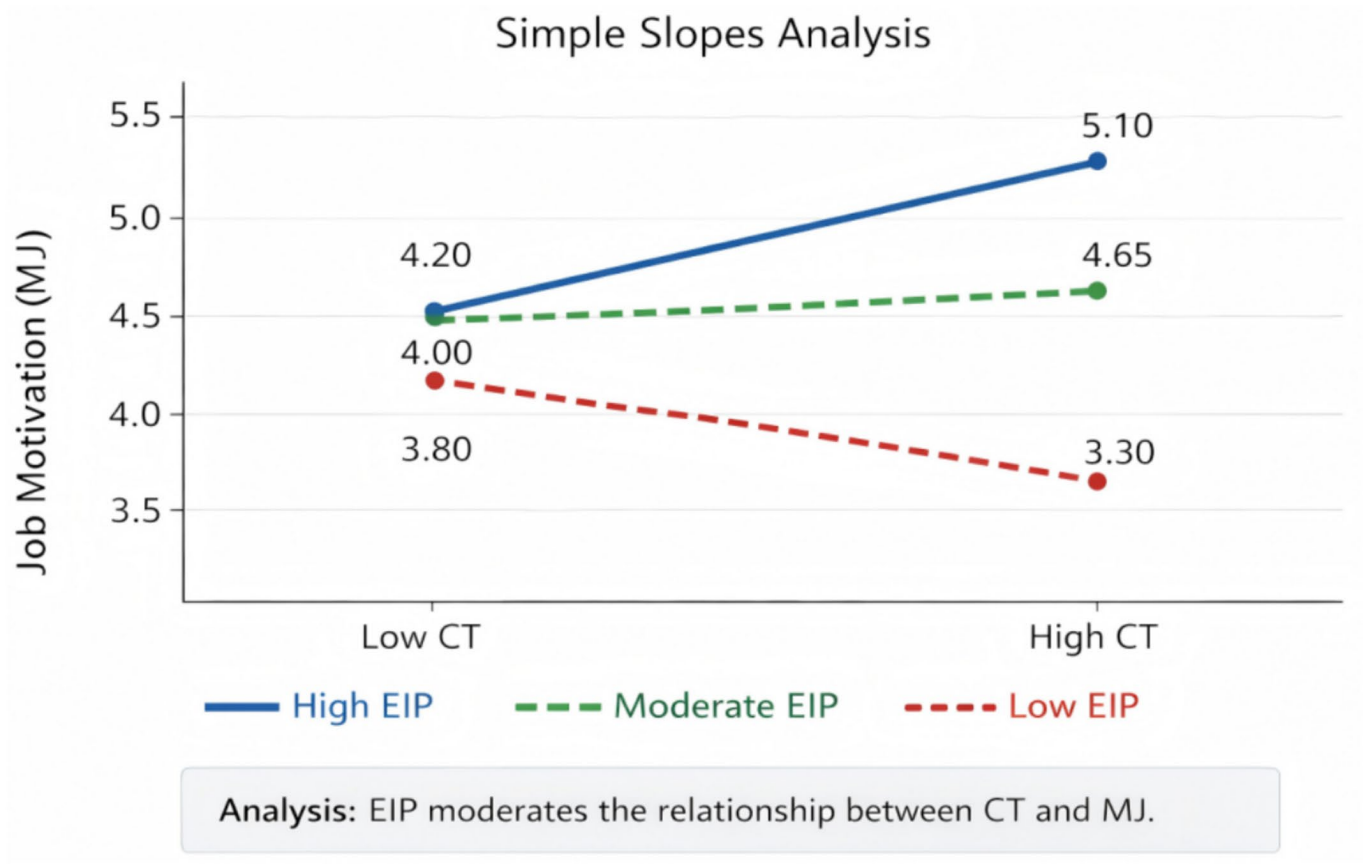
Simple slope plots of moderating effects.

Through a systematic examination of the results of the moderating effect analysis, we can gain a deeper understanding of the boundary role played by emotional information tendency in the process of critical thinking and emotional education influencing ethical judgment. As shown in Table 6, the pre-test moral judgment, as a basic variable, exhibits a standardized coefficient of 0.59, once again highlighting the crucial influence of initial moral level. As depicted in Figure 9, the main effect coefficients of critical thinking and emotional education are 0.21 and 0.24, respectively, further verifying the positive effects of these two abilities on enhancing ethical judgment. To present the moderating pattern more intuitively, this study further adds simple slope plots (Figure 9) to display the effects of critical thinking and emotional education on ethical judgment under low and high levels of post-truth sensitivity. The results show that both interventions have stronger positive effects when post-truth sensitivity is low, whereas the slopes become noticeably weaker when post-truth sensitivity is high, indicating that reliance on emotionalized information weakens the practical effectiveness of the dual-dimensional intervention. The emotional information tendency itself exhibits a negative impact of −0.17, indicating its inhibitory effect on ethical judgment. After introducing the moderating effect, the study found that emotional information tendency significantly weakens the positive effects of critical thinking and emotional education, with moderating coefficients of −0.13 and −0.16, respectively, suggesting that the higher an individual’s dependence on emotional information, the more likely the effect of educational intervention will be diminished. In terms of the change in model explanatory power, the introduction of the moderating effect has increased the model’s explanatory power by 4%. Although the increase is not significant, it is statistically significant, confirming the importance of considering individual differences. After controlling for all variables, the main effect coefficient of emotional education remains at 0.20, indicating a certain stability in its influence. These findings not only deepen our understanding of the formation mechanism of ethical judgment but also provide empirical evidence for developing differentiated educational strategies for students with different information processing tendencies. Overall, the moderating effect analysis reveals the boundary conditions of the effectiveness of educational intervention, providing important insights for improving the cultivation model of ethical judgment for college students.

**Table 6:** Analysis results of moderating effect (*N* = 600).

Predictor variable	Model 1 (β)	Model 2 (β)	Model 3 (β)
Control variable			
Pre-test MJ	0.59***	0.57***	0.55***
Main effect			
CT	0.21***	0.19***	0.18***
EE	0.24***	0.22***	0.20***
EIP	−0.17**	−0.15**	−0.14*
Moderating effect			
CT × EIP	–	−0.13*	−0.12*
EE × EIP	–	−0.16**	−0.15**
*R* ^2^	0.52	0.56	0.58
Δ*R*^2^	–	0.04**	0.02

Through systematic analysis of main effects and moderating effects, the study found that critical thinking and emotional education have a significant positive impact on college students’ ethical judgment, confirming the rationality of the dual-dimensional model. More importantly, emotional information tendency exhibits a significant moderating effect in the relationship between the two, indicating that an individual’s sensitivity to emotional information significantly affects the actual effectiveness of educational interventions. This finding not only expands the existing theoretical understanding of the formation mechanism of ethical judgment but also provides important insights for the development of differentiated educational strategies. The research results also show that the main effects exhibit significant differences under different levels of moderation, further verifying the necessity of considering individual traits in educational interventions. Overall, these analytical results provide a solid empirical foundation for improving the cultivation mechanism of college students’ ethical judgment and also point out new directions for future research.

### 4.4 Robustness test

Robustness testing is a crucial step in ensuring the reliability of research conclusions. Sub-sample analysis and testing methods can effectively evaluate the applicability and stability of research models across different groups. This chapter employs a grouped regression approach, dividing the total sample based on key characteristics such as gender, major, and grade, to examine whether the main effects and moderating effects remain consistent across different sub-groups. This method not only verifies the universality of research findings but also reveals the impact of potential group differences on research conclusions. Sub-sample analysis provides empirical support for the robustness of research conclusions by comparing the stability of model parameters across different groups. This testing approach effectively controls the interference caused by sample heterogeneity, ensuring that research conclusions do not deviate due to the characteristics of specific sub-groups. By systematically examining the performance of research models in different sub-samples, it can provide a more comprehensive evidence base for the external validity of research conclusions.

**Figure 10:**
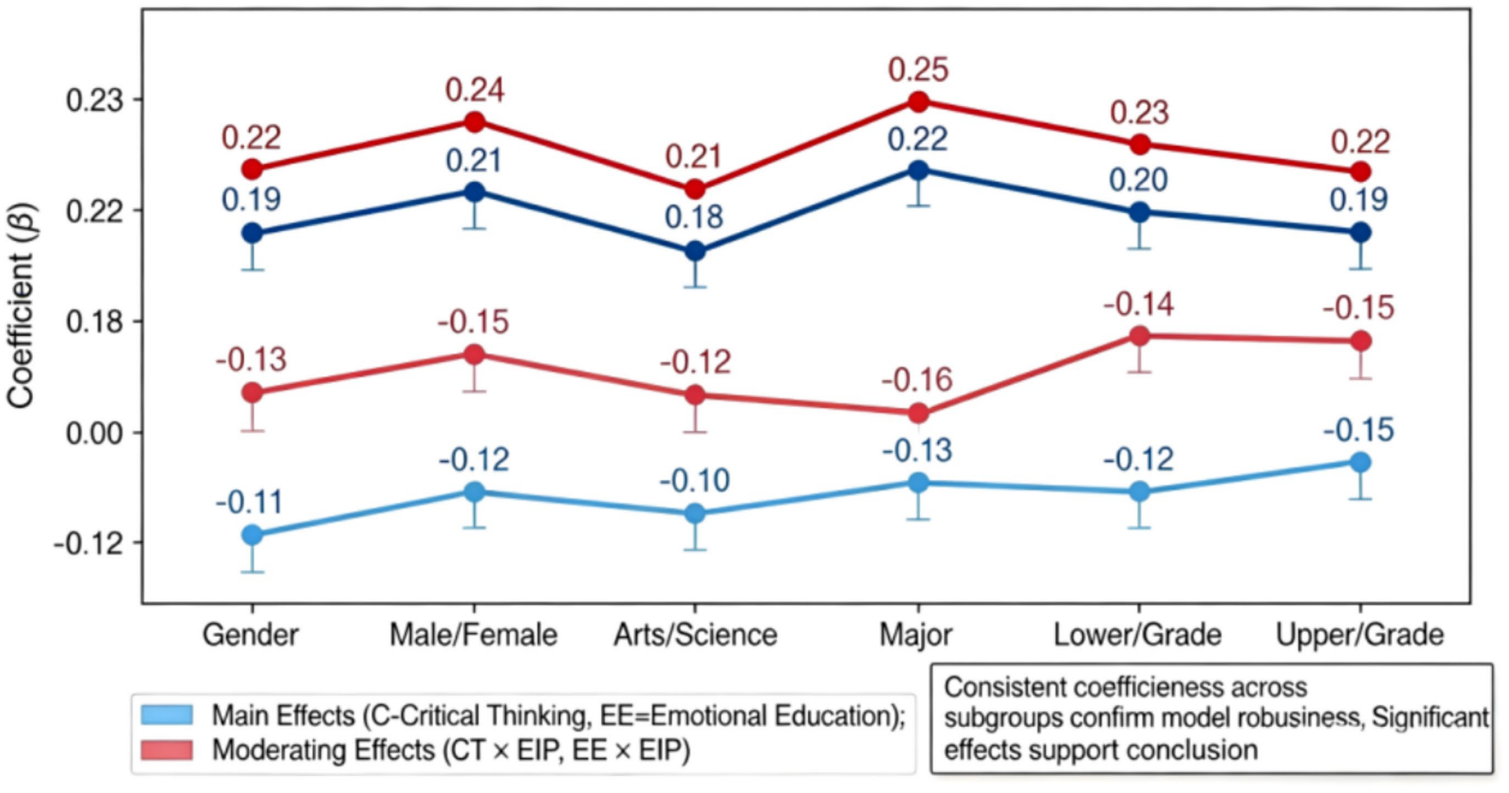
Sub-sample analysis result chart.

Through systematic examination of the analysis results of sub-samples, the universality and stability of research conclusions across different groups can be comprehensively evaluated. As shown in Table 7, in terms of gender, both male and female sub-samples demonstrate a significant promotional effect of critical thinking and emotional education on ethical judgment, with standardized coefficients reaching 0.19 and 0.22, respectively. Furthermore, the moderating effect of emotional information tendency exhibits a negative impact in both groups, confirming the robustness of the research model at the gender level. As illustrated in Figure 10, the grouping analysis based on professional background reveals that the main effect coefficients for liberal arts and science students are 0.18 and 0.22, respectively. Although the effect size is slightly higher for science students, both groups maintain statistical significance, and the direction of the moderating effect is completely consistent, indicating that professional differences do not alter the core conclusions. The results of grade grouping are equally persuasive, with the main effect coefficients for lower and upper grade students being 0.20 and 0.19 respectively, and the moderating effect coefficients remaining highly similar, suggesting that the effect of educational intervention does not undergo essential changes due to grade differences. The direction of the moderating effect in all sub-samples is consistent with that in the full sample analysis, and the vast majority reaches a statistically significant level, further enhancing the credibility of the research conclusions. These findings not only validate the applicability of the dual-dimensional model across different groups but also provide important references for differentiated strategies in educational practice.

**Table 7:** Sub-sample analysis results (*N* = 600).

Grouping variable	Subsample	Sample size	Main effect (β)	Moderation effect (β)
Gender	Male	300	CT:0.19** < br > EE:0.22**	CT × EIP:-0.11* < br > EE × EIP: –0.13*
	Woman	300	CT:0.21** < br > EE:0.24**	CT × EIP:-0.12* < br > EE × EIP: –0.15**
Major	Liberal arts	320	CT:0.18** < br > EE:0.21**	CT × EIP:-0.10 < br > EE × EIP: –0.12*
	Science	280	CT:0.22** < br > EE:0.25**	CT × EIP:-0.13* < br > EE × EIP: –0.16**
Grade	Lower grades	290	CT:0.20** < br > EE:0.23**	CT × EIP:-0.11* < br > EE × EIP: –0.14*
	Senior grade	310	CT:0.19** < br > EE:0.22**	CT × EIP:-0.12* < br > EE × EIP: –0.15**

*p < 0.05, **p < 0.01.

The results of the sub-sample analysis indicate that the research model exhibits good stability across different gender, major, and grade groups. The direction and intensity of the main effect and moderating effect are basically consistent with those found in the full-sample analysis. This finding confirms that the research conclusions have high internal validity, indicating that the promotion effect of critical thinking and emotional education on ethical judgment, as well as the moderating effect of emotional information tendency, are prevalent across different groups. Although there are slight differences in effect sizes among some sub-groups, these differences do not reach a statistically significant level, further strengthening the reliability of the research conclusions. It should also be noted that the post-grouping subsample sizes were mainly used for robustness comparison and consistency checks, and their statistical power for detecting relatively weak interaction effects remains limited. Therefore, the grouped moderation results are interpreted with caution, and future research is encouraged to improve the power of interaction tests by expanding the sample across regions or combining data from multiple universities. The sub-sample analysis also found that the research model exhibits similar predictive patterns among liberal arts and science students, indicating that differences in professional background do not have a substantial impact on the research conclusions. Overall, the results of the robustness test provide strong support for the research conclusions, enhance the theoretical value and practical guidance of the research findings, and provide empirical evidence for the subsequent promotion and application of the research in different groups.

## 5 Discussion

The present study provides empirical support for the educational value of integrating critical thinking and emotional education in the cultivation of college students’ ethical judgment. Rather than treating ethical judgment as the outcome of a single instructional factor, the findings suggest that it is more appropriately understood as a coordinated process in which cognitive discipline and affective engagement jointly shape students’ evaluative responses in ethically complex contexts. In this respect, the stronger performance of the dual-dimensional intervention group, relative to both the single-dimensional groups and the control group, is better interpreted not simply as evidence of effectiveness, but as support for a more integrated account of ethical judgment formation.

This interpretation is broadly consistent with earlier work showing that cognitive training, emotional education, and their interaction can promote moral and ethical judgment, while also extending these discussions into the context of complex information environments (Myyry et al., 2010; Durlak et al., 2011; Scuotto et al., 2024). What emerges more clearly from the present findings is that the contribution of the dual-dimensional model lies not only in combining two educational components, but in offering a more context-sensitive explanatory framework. Ethical judgment appears to depend not only on students’ reasoning capacity or emotional responsiveness in isolation, but also on the informational conditions under which judgment is formed.

The pattern of results further suggests a differentiated yet complementary mechanism. Critical thinking training was more closely associated with improvement in the cognitive dimension of ethical judgment, whereas emotional education showed a stronger connection with the emotional dimension. When these two components were combined, the intervention produced more comprehensive gains than either single-track approach alone. This lends support to the view that reasoning and emotion should not be treated as competing explanatory routes, but as mutually reinforcing conditions for ethical judgment in the post-truth era. Within this framework, post-truth sensitivity becomes particularly important. The moderating analysis indicates that intervention effects are partly shaped by students’ sensitivity to emotionally charged and weakly verified information, which is in line with research on emotionalized information reliance, difficulties in assessing veracity, and judgmental bias in post-truth settings (Pennycook and Rand, 2021; Maertens et al., 2024).

The discussion also carries implications for ethics education in higher education. If ethical judgment is shaped by both analytical and affective processes, then ethics education should move beyond the transmission of normative knowledge alone and pay greater attention to students’ capacity to make judgments under conditions of informational ambiguity. The findings suggest that integrated rather than isolated instructional design may be more appropriate, particularly for students who are more vulnerable to post-truth influences. At the same time, although the robustness checks across gender, major, and grade provide some support for the internal stability of the findings, such consistency should not be taken as equivalent to broader external validity across regions or institutional settings. To make this contribution more transparent, Table 8 summarizes the key differences between the present study and prior research, particularly with regard to the dual-dimensional design and the moderating role of post-truth sensitivity.

**Table 8:** Comparison table of previous studies.

Research dimension	Previous research mainly found	The main findings of this study are
The role of critical thinking	Effect size *d* = 0.40 (single-dimensional intervention)	Effect size *d* = 0.42 (two-dimensional intervention)
The role of emotional education	Effect size *d* = 0.35 (unidimensional intervention)	Effect size *d* = 0.38 (two-dimensional intervention)
Dual-dimensional synergistic effect	Unquantified	The effect size *d* = 0.75, significantly superior to single-dimensional intervention
The impact of post-truth sensitivity	Lack of empirical data	Significantly negative regulation (*β* = −0.21, *p* < 0.05)
Innovativeness of intervention design	Mostly single-dimensional intervention	Propose and validate a dual-dimensional collaborative model

### 5.1 Educational implications

The present findings suggest that ethics education in higher education should adopt a more integrated approach. Since ethical judgment appears to be shaped by both cognitive discipline and affective engagement, instructional design may be more effective when critical thinking training and emotional education are combined rather than separated. In the context of post-truth information environments, ethics education should go beyond the transmission of moral principles alone and place greater emphasis on students’ ability to evaluate emotionally charged and weakly verified information. The results also imply that students with higher post-truth sensitivity may require more differentiated support. Overall, the proposed framework may offer a useful basis for developing ethics education that is more responsive to informational complexity in contemporary university settings.

### 5.2 Limitations

Several limitations should be considered when interpreting the present findings. The sample was drawn primarily from universities within a relatively specific regional context, and the conclusions should therefore be understood within that empirical scope. Although the subgroup analyses across gender, major, and grade support the internal stability of the findings to some extent, such consistency does not automatically imply broader external generalizability across different institutional types, regional settings, or cultural contexts. The present study is therefore better regarded as evidence from a defined higher-education environment rather than as a universally applicable account of ethical judgment intervention.

The measurement strategy also calls for caution. Ethical judgment was assessed through standardized scales and situational tests, which provide a useful but still partial representation of students’ ethical decision-making capacities. While this approach improves analytical comparability, it cannot fully capture the complexity of ethical behavior in naturalistic settings, where social expectations, institutional norms, and contextual pressures may shape judgment differently from test-based responses.

The time span of the intervention and follow-up should also be taken into account. The present findings are more appropriately interpreted as evidence of short- to medium-term educational effects than as confirmation of long-term developmental change. Future research may therefore benefit from testing the proposed framework in more diverse educational settings, across broader regional and cultural contexts, and over longer periods of observation.

## 6 Conclusion

This study empirically verified the effectiveness of the dual-dimensional model of critical thinking and emotional education in enhancing college students’ ethical judgment. The research findings indicate that, compared to single-dimensional intervention methods, the dual-dimensional model integrating cognitive training and emotional cultivation yields a more significant improvement effect. This discovery provides an important theoretical basis for the reform of ethical education in universities. The study found that post-truth sensitivity, as an important moderating variable, significantly impacts the effectiveness of educational interventions, suggesting that educational practice needs to fully consider individual differences among students. Therefore, the practical significance of this study lies not only in showing that the dual-dimensional approach outperforms the single-dimensional one, but also in suggesting that university ethical education should adopt an implementation logic of “stratified identification–differentiated intervention–dynamic adjustment.” In particular, more targeted instructional support should be provided for highly sensitive groups so as to improve the effectiveness and sustainability of educational resource allocation. The study employed rigorous experimental design and multivariate statistical methods to ensure the scientificity and reliability of the research conclusions, and also verified the stability of the research results across different groups through robustness tests. The main contribution of the study lies in constructing and validating an ethical education model that integrates cognitive and emotional dimensions, breaking through the limitations of traditional single-dimensional research. By revealing the synergistic mechanism of critical thinking and emotional education, this study provides a new theoretical perspective for understanding the cultivation path of ethical judgment in the post-truth era. The research results have direct reference value for curriculum design and teaching method reform in universities, especially providing empirical support for balancing rational thinking and emotional experience. These findings provide important theoretical support and practical insights for addressing the challenges of ethical education in the contemporary information environment.

## Data Availability

The original contributions presented in the study are included in the article/supplementary material, further inquiries can be directed to the corresponding author.
